# Influence of the compatible solute sucrose on thylakoid membrane organization and violaxanthin de-epoxidation

**DOI:** 10.1007/s00425-021-03699-w

**Published:** 2021-08-15

**Authors:** Reimund Goss, Christian Schwarz, Monique Matzner, Christian Wilhelm

**Affiliations:** 1 Institute of Biology, Department of Plant Physiology, Leipzig University, Johannisallee 21-23, 04103 Leipzig, Germany; 2 German Centre for Integrative Biodiversity Research (iDiv) Halle-Jena-Leipzig, Deutscher Platz 5e, 04103 Leipzig, Germany

**Keywords:** Lipid domain, Membrane organization, MGDG, Non-bilayer lipid phase, Violaxanthin de-epoxidase, Xanthophyll cycle

## Abstract

**The compatible solute sucrose reduces the efficiency of the enzymatic de-epoxidation of violaxanthin, probably by a direct effect on the protein parts of violaxanthin de-epoxidase which protrude from the lipid phase of the thylakoid membrane.**

The present study investigates the influence of the compatible solute sucrose on the violaxanthin cycle of higher plants in intact thylakoids and in in vitro enzyme assays with the isolated enzyme violaxanthin de-epoxidase at temperatures of 30 and 10 °C, respectively. In addition, the influence of sucrose on the lipid organization of thylakoid membranes and the MGDG phase in the in vitro assays is determined. The results show that sucrose leads to a pronounced inhibition of violaxanthin de-epoxidation both in intact thylakoid membranes and the enzyme assays. In general, the inhibition is similar at 30 and 10 °C. With respect to the lipid organization only minor changes can be seen in thylakoid membranes at 30 °C in the presence of sucrose. However, sucrose seems to stabilize the thylakoid membranes at lower temperatures and at 10 °C a comparable membrane organization to that at 30 °C can be observed, whereas control thylakoids show a significantly different membrane organization at the lower temperature. The MGDG phase in the in vitro assays is not substantially affected by the presence of sucrose or by changes of the temperature. We conclude that the presence of sucrose and the increased viscosity of the reaction buffers stabilize the protein part of the enzyme violaxanthin de-epoxidase, thereby decreasing the dynamic interactions between the catalytic site and the substrate violaxanthin. This indicates that sucrose interacts with those parts of the enzyme which are accessible at the membrane surface of the lipid phase of the thylakoid membrane or the MGDG phase of the in vitro enzyme assays.

## Introduction

The violaxanthin (V) cycle represents an important photoprotection mechanism of higher plants and algae (Goss and Lepetit 2015). It plays an important role in the structural re-arrangement of the light-harvesting complex of photosystem II (LHCII), which leads to the dissipation of excess excitation energy as heat during high light illumination. This process has been termed non-photochemical quenching of chlorophyll (Chl) a fluorescence, NPQ (Ruban 2016).

The V cycle consists of a forward reaction which is typically driven by high light illumination. It comprises the stepwise de-epoxidation of the xanthophyll V, which contains two epoxy groups, to the mono-epoxide antheraxanthin (A) and finally to the epoxy-free zeaxanthin (Z). The forward reaction of the V cycle is catalysed by the enzyme V de-epoxidase (VDE). During low light or darkness the cycle is reverted and the two epoxy groups are re-introduced by the enzyme Z epoxidase (ZEP).

VDE is a nuclear-encoded protein and, like the ZEP, belongs to the lipocalin family of proteins (Hieber et al. 2000). Lipocalins are diverse proteins which bind hydrophobic substrates and usually act as carrier proteins. VDE has a pH-optimum of pH 5.2 (Pfündel et al. 1994) and utilizes ascorbate as co-substrate to reduce the epoxy group before its abstraction as H_2_O. In contrast to ZEP, which seems to be permanently associated with the stromal side of the thylakoid membrane as peripheral membrane protein (Schaller et al. 2012), VDE only attaches to the luminal side of the membrane during the operation of the V cycle (Hager and Holocher 1994). Binding of VDE to the membrane takes place after its pH-dependent activation which may comprise the dimerization of inactive VDE monomers (Arnoux et al. 2009; Hallin et al. 2016).

It has been suggested that VDE binds to non-bilayer lipid phases which are enriched in the main thylakoid membrane lipid monogalactosyldiacylglycerol (MGDG; Goss et al. 2007). According to recent models (Jahns et al. 2009; Goss and Lepetit 2015; Garab et al. 2016; Goss and Latowski 2020), the non-bilayer or inverted hexagonal phases (H_II_-phases) are formed when LHCII dissociates from the PSII core complex and forms aggregates during the establishment of non-photochemical quenching of chlorophyll a fluorescence (NPQ). Aggregation of LHCII leads to a segregation of MGDG which in darkness or low light interacts with the antenna complexes and through this interaction adopts a bilayer structure. Due to the special nature of the MGDG molecule as a non-bilayer forming lipid, segregation and local enrichment of MGDG supports the formation of non-bilayer lipid phases within or attached to the thylakoid bilayer. Results obtained with artificial membrane systems and isolated lipids have suggested that the enzymatic de-epoxidation of V to A and Z takes place in the non-bilayer lipid phase (Goss et al. 2005, 2007), which, in the native membrane, would require the detachment of V molecules from their binding sites at the LHC apoproteins followed by diffusion into the lipid phase. This idea is supported by results from experiments with isolated, exogenous VDE which show that the de-epoxidation reaction is also possible if the VDE is not located at the luminal but at the stromal side of the thylakoid membrane (Arvidsson et al. 1997; Macko et al. 2002). Since in these experiments the VDE located at the stromal side of the membrane, in contrast to the native VDE at the luminal side, had no access to the stacked grana membranes where V bound to the LHCII is located, it was concluded that de-epoxidation is only possible in a lipid phase after diffusion of the V cycle pigments. This special lipid phase might even be located in a part of the membrane which shows a certain distance to the LHC proteins. However, other studies have pointed to a closer association between the non-bilayer phase and the antenna proteins. It has been shown that the isolation of LHCII associated with an MGDG shield enriched in V cycle pigments is possible (Schaller et al. 2010) and recently, the purification of an active V cycle domain consisting of LHCII, MGDG, V and bound VDE has been reported (Goss et al. 2017). Analysis of the molecular structure of LHCII at 2.72 Å resolution has indicated the existence of a putative VDE binding site at the LHCII apoprotein (Liu et al. 2004), which also argues for a close association or even a direct interaction between the VDE and the LHCII.

The localization of the non-bilayer lipid phase is still an open question. It has been proposed that non-bilayer phases exist or are established within the plane of the thylakoid membrane (Jahns et al. 2009). Other authors suggest that the non-bilayer or H_II_-phases are excluded from the membrane bilayer to the thylakoid lumen (Garab et al. 2016). Irrespective of a localization within or outside of the membrane bilayer it is clear that the non-bilayer phases have to remain in tight local and functional association with the rest of the thylakoid membrane.

The formation of extensive H_II_-phases in higher plant thylakoids can be triggered by certain artificial conditions. Exposition of isolated thylakoid membranes to short periods of high temperatures above 45 °C (Gounaris et al. 1984) or pH values below 4.5 (Thomas et al. 1985) lead to lipid phase separation and irreversible formation of non-bilayer lipid structures. In addition, incubation of isolated thylakoids in reaction buffers complemented with high concentrations of compatible solutes, such as sucrose, trehalose, sorbitol or betaine, induces the phase separation of non-bilayer forming lipids (Williams et al. 1992). These earlier results, which demonstrate the existence of non-bilayer lipid phases by freeze-fracture electron microscopy, are supported by recent results. With the help of ^31^P-NMR spectroscopy and the fluorescent dye merocyanine-540 it was shown that in spinach thylakoids one bilayer and up to three non-bilayer phases may co-exist (Garab et al. 2017). In the same study it was demonstrated that the formation of non-bilayer phases is sensitive to osmolytes, i.e. compatible solutes, and the presence of a low pH.

It is the intention of the present study to analyse the influence of non-bilayer lipid phases on the efficiency of V de-epoxidation in the native thylakoid membrane. In the present experiments formation of non-bilayer lipid phases in thylakoids should be triggered by the presence of high concentrations of the compatible solute sucrose. Taking into account the importance of non-bilayer lipid phases for efficient V de-epoxidation, the induction of extensive H_II_-phases by the compatible solute is thought to stimulate the conversion of V to A and Z. To determine if sucrose, besides its influence on thylakoid membrane structure, exerts a more direct effect on the enzyme VDE, in vitro de-epoxidation assays with the isolated VDE in the presence or absence of sucrose are additionally performed. In the present experiments the organization of the lipid phase of the thylakoid membrane, as a possible indicator of lipid phase separation, is monitored via the fluorescence emission spectrum of Laurdan. Laurdan is a fluorescent dye which can provide information about the membrane order and the membrane phase properties (Parasassi et al. 1990, 1991, 1998; Hellgren 1996; Szilagyi et al. 2008). The Laurdan molecule is incorporated into the membrane with the fluorescent group located at the level of the glycerol backbone and the fatty acid tail incorporated into the hydrophobic core of the membrane. Laurdan is sensitive to the polarity and the dipoles in the surrounding lipid phase and thus to the hydration level in its environment. Laurdan shows different fluorescence emission spectra when incorporated into artificial phospholipid membranes in the gel and liquid crystalline phases, respectively. The red-shift of the fluorescence emission in membranes in the liquid crystalline phase has been attributed to an increased concentration of water molecules at the hydrophilic/hydrophobic membrane interface. Although the shift of the membrane from the ordered gel phase to the more disordered liquid crystalline phase is accompanied by an increase of the membrane fluidity, Harris et al. (2002) demonstrated that the spectral shift of Laurdan can be observed independently from changes in the fluidity and that Laurdan mainly detects changes in the order of the membrane lipids. With respect to the analysis of the V cycle in the present study, the efficiency of V de-epoxidation is monitored in V de-epoxidation assays with the isolated thylakoid membranes and VDE, respectively, in the presence or absence of the compatible solute sucrose.

## Materials and methods

### Plant material

Fresh spinach (*Spinacia oleracea*) leaves were obtained from the local market.

### Isolation of thylakoid membranes, violaxanthin de-epoxidase and pure violaxanthin

Intact thylakoid membranes were isolated according to Jensen and Bassham (1966). VDE was prepared from the isolated thylakoids by seven freeze–thaw cycles according to Hager and Holocher (1994). After the preparation VDE was diluted five times with preparation buffer and aliquots were stored at − 20 °C. V was purified from the isolated thylakoids by HPLC according to the procedure described by Goss (2003).

### General measurement conditions

The measurements were performed with intact thylakoid membranes or purified VDE in different reaction buffers. The buffers were either free of sucrose or contained sucrose at a concentration of 1 or 2 M. All reaction buffers were adjusted to pH 5 to allow V de-epoxidation in the dark. All buffers contained 10 mM KCl, 5 mM MgCl_2_, and 40 mM MES. All measurements were performed at temperatures of 30 and 10 °C, respectively. The measurements were repeated at least three times with independent thylakoid or VDE preparations.

### Violaxanthin de-epoxidation assays

V de-epoxidation assays were performed with the isolated thylakoid membranes and the purified VDE. For the de-epoxidation assays with isolated thylakoids the thylakoid membranes were pre-incubated in reaction buffer without sucrose for 5 min at 30 °C. The pre-incubation at 30 °C was chosen to allow the VDE to completely bind to the thylakoid membrane before the start of the de-epoxidation reaction. For the pre-incubation a Chl concentration of 200 µg mL^−1^ was chosen, after the pre-incubation the thylakoid membranes were diluted with the different reaction buffers to obtain a final Chl concentration of 20 µg mL^−1^. The reaction buffers were adjusted to either 30 or 10 °C. The mixture was then incubated for a further 5 min at 30 or 10 °C. After a control sample had been taken, the V de-epoxidation was started by addition of reduced ascorbate with a final concentration of 30 mM. Samples were collected after 2, 5, 10 and 30 min of the de-epoxidation reaction.

The de-epoxidation assays with the purified VDE were performed according to the protocol originally published by Yamamoto and Higashi (1978). Purified V and MGDG were mixed and added to the different reaction buffers which were adjusted to either 30 or 10 °C. The final V and MGDG concentrations were 0.4 and 11.6 µM, respectively. After the addition of 125 µL of the diluted VDE solution the mixture was equilibrated for 5 min at 30 or 10 °C before a control sample was taken. V de-epoxidation by the purified VDE was then started by the addition of 30 mM ascorbate and samples were collected after 2, 5, 10 and 15 min of the enzyme assay.

### Pigment determination

Immediately after the collection of the samples in the V de-epoxidation assays, 750 µL of the sample were injected into Eppendorf tubes containing an equal volume of a pigment extraction medium which consisted of CHCl_3_:MeOH:NH_3_ in the ratio 1:2:0.004 (by vol.). For complete pigment extraction, the samples were repeatedly stirred and centrifuged for 2 min at 20.000 g and 4 °C (1–14 K, Sigma, Osterode, Germany). 200 µL of the lower organic phase were then collected, dried under a gentle stream of nitrogen and stored at − 80 °C until pigment analysis by HPLC was performed.

The dried pigments were dissolved in a medium consisting of 90% methanol/0.2 M ammonium acetate (9:1, v/v) and 10% ethyl acetate, centrifuged for 2 min at 20.000 g and 4 °C (1–14 K, Sigma) and analysed by HPLC (Ultimate 3000 HPLC, ThermoFisher, Waltham, MA, USA) using a Nucleosil ET 250/4 120-5 C18 column (Macherey–Nagel, Düren, Germany) according to Frommolt et al. (2001). Pigments were quantified according to Böhme et al. (2002).

To compare the efficiency of V de-epoxidation in the samples with or without sucrose the de-epoxidation state of the V cycle pigment pool (DES) was calculated using the following equation:

${\text{DES}}\; = \;\left( {0.5{\text{A}}\; + {\text{Z}}} \right)/\left( {{\text{V}}\; + \;{\text{A}}\; + {\text{Z}}} \right)$, with V—violaxanthin, A—antheraxanthin, Z—zeaxanthin.

De-epoxidation rates for V de-epoxidation in thylakoids and in vitro enzyme assays, respectively, were calculated as change of the DES per minute. For the calculation of the de-epoxidation rates at 30 °C the DES at 2 min of the de-epoxidation reaction was used from which the value at the beginning of the measurements, i.e. time point 0 min, was subtracted. For the measurements at 10 °C the de-epoxidation rates were calculated with the DES obtained after 5 min of the de-epoxidation assays. Again the de-epoxidation state at the beginning of the measurements was subtracted from the values detected after 5 min. The time point 2 min for the de-epoxidation assays at 30 °C was chosen because V de-epoxidation under these conditions exhibited rapid kinetics. For the determination of the de-epoxidation rates at 10 °C the time point 5 min was used because the de-epoxidation reaction at the lower temperature was significantly slower compared to that at 30 °C and after 2 min of the de-epoxidation reaction only minor changes of the DES could be detected. Since the de-epoxidation rates were calculated at fixed time points, and taking into account that the de-epoxidation rate changes over time, comparison of the de-epoxidation rates should be regarded as semi-quantitative.

### Laurdan fluorescence spectroscopy

For the Laurdan fluorescence measurements with isolated thylakoids, the thylakoid membranes were pre-incubated in the reaction buffer without sucrose. The pre-incubation was performed for 5 min at 30 °C with a Chl concentration of 25 µg mL^−1^ and a Laurdan (6-dodecanoyl-2-dimethylaminonaphthalene, Sigma-Aldrich, Taufkirchen, Germany) concentration of 10 µM. The pre-incubation conditions were chosen to enable a complete penetration of Laurdan molecules into the lipid bilayer of the thylakoid membranes. After the pre-incubation the thylakoids were diluted with the different reaction buffers described above to yield final Chl and Laurdan concentrations of 2.5 µg mL^−1^ and 1 µM, respectively. The reaction buffers were either adjusted to 30 or 10 °C. The fluorescence measurements were started 2 min after the final dilution and were performed at either 30 or 10 °C. Laurdan fluorescence emission was excited at 390 nm and was recorded in a wavelength range from 400 to 700 nm in a FluoroMax-4 spectrofluorometer (Jobin–Yvon Horiba, Longjumeau, France). For both the excitation and the emission light bandwidths of 5 nm were used.

To obtain information about the state of the MGDG phase in the enzyme assays, which were carried out with the purified VDE, additional Laurdan fluorescence spectra were recorded. In this case stock solutions of MGDG and Laurdan were carefully mixed for 2 min with a pipette tip to obtain a final MGDG to Laurdan ratio of 116:1. After the incubation the different reaction buffers were added to obtain final MGDG and Laurdan concentrations of 11.6 µM and 0.1 µM, respectively. The reaction buffers were adjusted to either 30 or 10 °C. After a further incubation of 5 min the Laurdan fluorescence spectra were recorded with the parameters described above.

To compare the organization of the lipid phase of thylakoid membranes in the presence or absence of compatible solutes the generalized polarization (GP) values (Parasassi et al. 1990) were calculated from the Laurdan fluorescence spectra using the following equation:

${\text{GP}}\; = \;\left( {{\text{I}}_{450} - {\text{I}}_{500} } \right)/\left( {{\text{I}}_{450} { + }\;{\text{I}}_{500} } \right)$, with I_450_—fluorescence emission maximum at 450 nm, I_500_—fluorescence emission maximum at 500 nm.

In artificial phospholipid membranes, the Laurdan fluorescence emission maximum at 450 nm represents the membrane in the higher ordered, so-called gel phase, whereas the emission maximum at 490–500 nm stands for a membrane in the less ordered state, the so-called liquid crystalline phase (Parassasi et al. 1991, 1998). To minimize interference with endogenous thylakoid fluorescence (Hellgren 1996), in the present measurements the emission at the longer wavelength of 500 nm was used to calculate the GP-values. The emission wavelengths of 450 and 500 nm were also used to determine the GP-values of the MGDG phase in the different reaction buffers used for the enzyme assays. Since especially the thylakoid membrane represents a much more complex system than artificial membranes composed of phospholipids, changes of the GP-values in the present study are interpreted very cautiously and only general changes of the membrane lipid phase organization and the order of membrane lipids are considered.

## Results

### Laurdan fluorescence spectroscopy

**Fig. 1:**
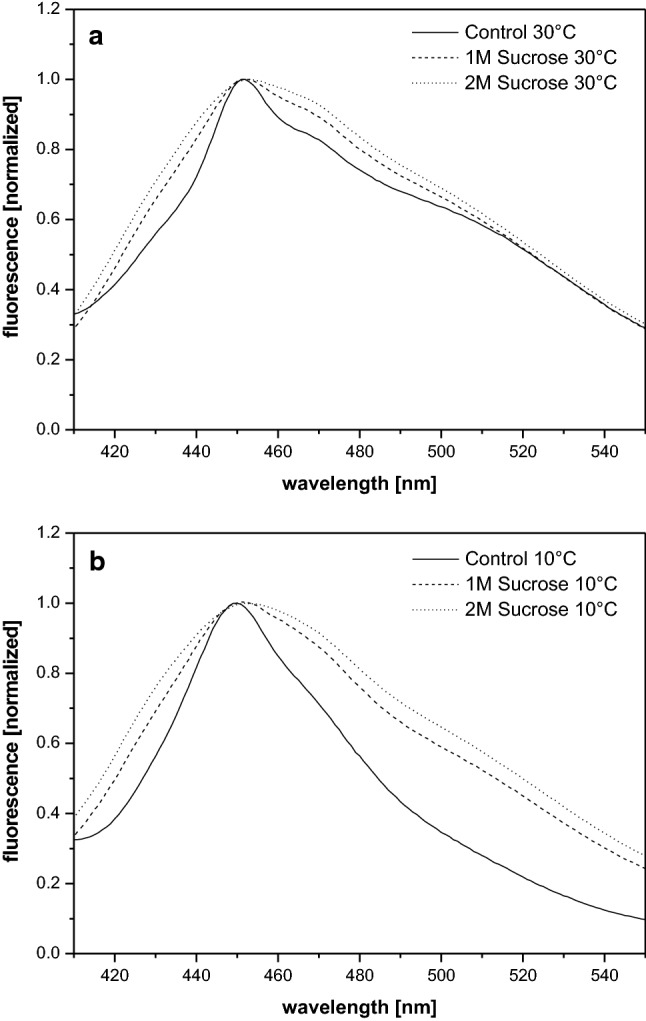
Fluorescence emission spectra of Laurdan incorporated into isolated thylakoid membranes diluted in reaction buffers without or with the compatible solute sucrose. Incubation and recording of the spectra was performed at temperatures of 30 °C (**a**) and 10 °C (**b**), respectively. The emission spectra were normalized to the emission maximum at 450 nm. Thylakoid membranes were used with a final Chl concentration of 2.5 µg mL^−1^, the final Laurdan concentration was 1 µM. The excitation wavelength for the Laurdan fluorescence was set to 390 nm. For further measurement conditions, including the pre-incubation conditions, see Materials and methods

**Fig. 2:**
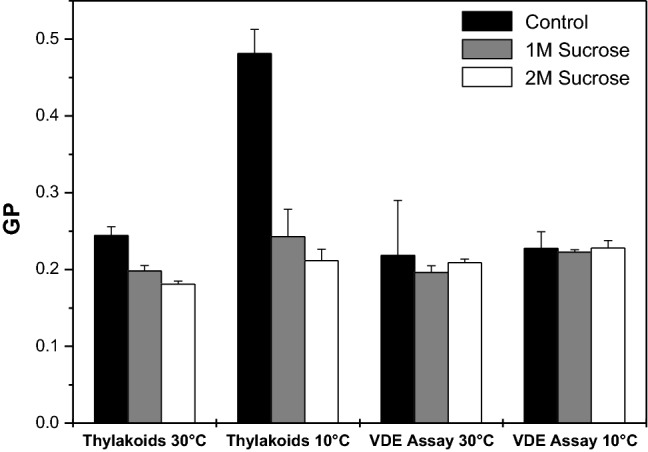
GP-values of Laurdan incorporated into thylakoid membranes or the MGDG phase of the in vitro enzyme assays at different sucrose concentrations and temperatures of 30 and 10 °C, respectively. The GP-values were derived from Laurdan fluorescence emission spectra as depicted in Figs. 1 and 3. For the calculation of the GP-values, the Laurdan emission maxima at 450 and 500 nm were used. For further details on the calculation of the GP-values see Materials and methods. Mean values of four independent measurements with the respective standard deviations are shown

The fluorescence emission spectrum of Laurdan incorporated into thylakoid membranes diluted in reaction buffer without sucrose at 30 °C exhibits a maximum at around 450 nm (Fig. 1a). It is further characterized by a pronounced shoulder in the fluorescence emission in the wavelength range from 450 to 520 nm. Thylakoids in the reaction buffer without sucrose at 30 °C are characterized by low GP-values of around 0.25 (Fig. 2). Dilution of isolated thylakoids into reaction buffers containing 1 and 2 M sucrose at 30 °C leads to a slight increase of the longer wavelength fluorescence contributions (Fig. 1a). The effect depends on the concentration of sucrose and more intense increases are observed at the higher sucrose concentration. The spectral changes lead to slight changes of the GP-values (Fig. 2) which depict a minor concentration-dependent decrease in the presence of sucrose. The mean of the GP-values with their respective standard deviations depicted in Fig. 2 also provides information about the reliability of the spectral changes described in this section.

The fluorescence emission spectrum of Laurdan incorporated into thylakoids in reaction buffer without sucrose at 10 °C (Fig. 1b) shows pronounced differences compared to the respective spectrum at 30 °C. While the spectrum at 10 °C still exhibits a clear maximum at 450 nm, the longer wavelength components up to 520 nm are strongly reduced. The spectral differences result in the calculation of high GP-values of around 0.5 for these membranes (Fig. 2). In the presence of sucrose in the reaction buffers the Laurdan fluorescence emission spectra recorded at 10 °C are characterized by a much higher long wavelength fluorescence emission (Fig. 1b). The spectra are even comparable to the Laurdan emission spectra recorded for the thylakoid membranes diluted in the sucrose containing reaction buffers at 30 °C (Fig. 1a). This is in line with the GP-values calculated for the thylakoid membranes incubated with sucrose at 10 °C (Fig. 2) which are comparable to the GP-values of the thylakoid membranes incubated in the different buffers at 30 °C.

**Fig. 3:**
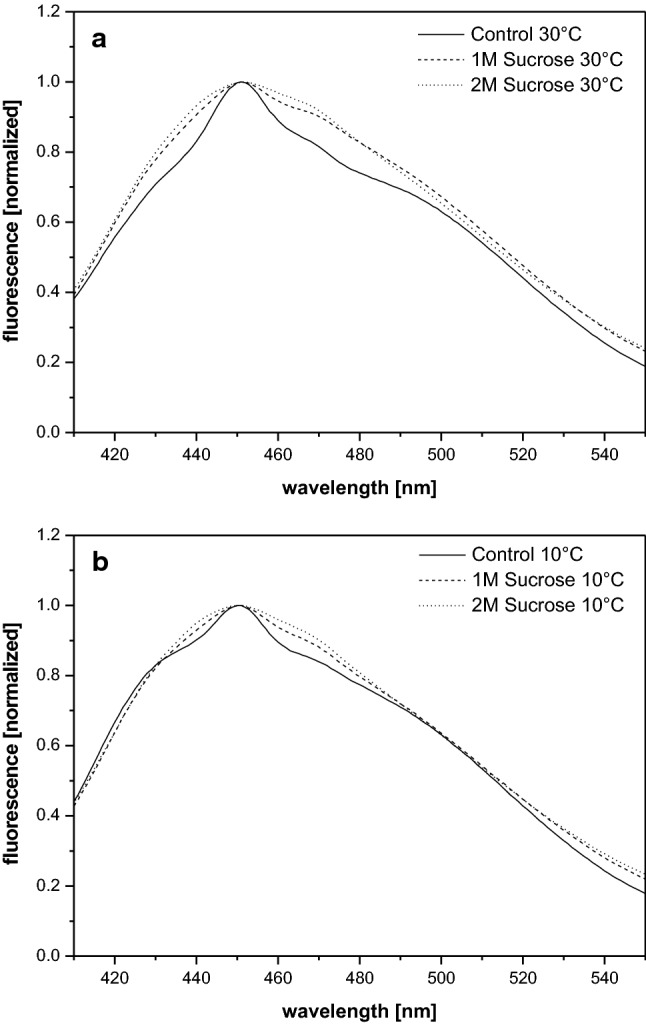
Fluorescence emission spectra of Laurdan incorporated into the MGDG phase of the in vitro enzyme assays performed in reaction buffers without or with sucrose. Incubation and recording of the spectra was performed at temperatures of 30 °C (**a**) and 10 °C (**b**), respectively. The emission spectra were normalized to the emission maximum at 450 nm. For further measurement conditions, including the pre-incubation conditions, see the legend of Fig. 1 and “Materials and methods”

The fluorescence emission spectra of Laurdan molecules incorporated into the MGDG phase of the in vitro enzyme assays (Fig. 3) show a comparable shape to the Laurdan spectra of isolated thylakoid membranes. The spectra are also characterized by a fluorescence emission maximum at around 450 nm and a pronounced fluorescence emission up to wavelengths of 520 nm. In contrast to the emission spectra of Laurdan incorporated into thylakoid membranes the lower temperature of 10 °C does not affect the emission spectra of Laurdan molecules in the MGDG phase of the enzyme assays significantly (Fig. 3b) and the spectra are comparable to those obtained at 30 °C (Fig. 3a). Only minor decreases in the long wavelength range from around 480 to 520 nm are observed and indicate a slight re-organization of the MGDG lipid phase. This is also corroborated by the GP-values which are slightly increased at the temperature of 10 °C compared to the values at 30 °C (Fig. 2). The presence of sucrose in the reaction buffers at 30 °C does not alter the Laurdan fluorescence emission spectra substantially. Only small increases of the emitted fluorescence can be observed in the wavelength range around the emission maximum at around 450 nm (Fig. 3a). This holds also true for emission spectra recorded for Laurdan molecules incorporated into the MGDG phases at 10 °C. In the presence of sucrose a minor increase of the fluorescence emission can be seen in the region of the emission maximum, the fluorescence emission at 500 nm is almost unchanged. The absence of a pronounced sucrose effect on the Laurdan fluorescence results in only very small decreases of the GP-values in the presence of sucrose, both at 30 and 10 °C (Fig. 2).

Summarizing the results of the Laurdan fluorescence spectroscopy it can be stated that in thylakoid membranes sucrose induces minor spectral changes of the Laurdan fluorescence emission at 30 °C. However, at 10 °C the effect of sucrose is very pronounced and a Laurdan fluorescence spectrum comparable to that of membranes at 30 °C can be observed. The MGDG phase in the in vitro enzyme assays seems to be more stable and both lower temperatures and high amounts of the compatible solute sucrose can induce only very minor changes of the organization of the lipid phase.

### V de-epoxidation

**Fig. 4:**
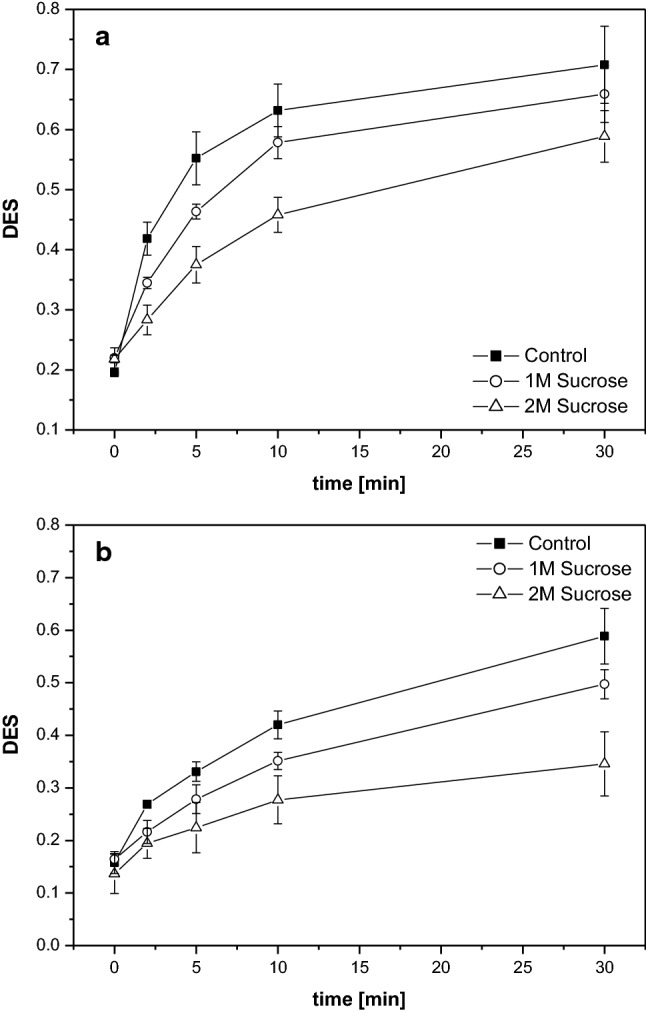
Time course of V de-epoxidation depicted as the de-epoxidation state of the V cycle pigment pool (DES). V de-epoxidation was performed at 30 °C (**a**) or 10 °C (**b**) in isolated thylakoid membranes diluted in reaction buffers without or with the compatible solute sucrose. Thylakoid membranes were used with a final Chl concentration of 20 µg mL^−1^, the co-substrate of VDE, ascorbate, was added at a final concentration of 30 mM. For further measurement conditions, including the pre-incubation conditions, see “Materials and methods”. Mean values of three independent measurements with the respective standard deviations are shown

Isolated thylakoid membranes diluted in the reaction buffer without sucrose exhibit a fast de-epoxidation of V to A and Z after the addition of ascorbate at 30 °C (Fig. 4a). The DES increases within minutes after the start of the reaction and after 5 to 10 min the largest part of V has been converted to Z. Dilution and incubation of thylakoid membranes in reaction buffers supplemented with sucrose leads to an inhibition of the V de-epoxidation reaction as evidenced by the slower increases of the DES during the time span of the present measurements (Fig. 4a). The inhibition of V de-epoxidation depends on the concentration of sucrose and a stronger inhibition is observed in the buffer containing 2 M sucrose compared to the reaction buffer with a sucrose concentration of 1 M.

The results of the thylakoid de-epoxidation assays performed at 10 °C with respect to the action of sucrose are similar to those obtained at 30 °C. Overall, V de-epoxidation is slower at the lower temperature and 30 min of reaction time lead to a lower DES compared to the DES obtained after 5 to 10 min of the reaction assays at 30 °C (Fig. 4b). As it is observed for the de-epoxidation assays at 30 °C, sucrose leads to a significant reduction of V de-epoxidation in a concentration-dependent manner, i.e. the higher concentration of sucrose leads to a stronger inhibition of the DES increase.

**Fig. 5:**
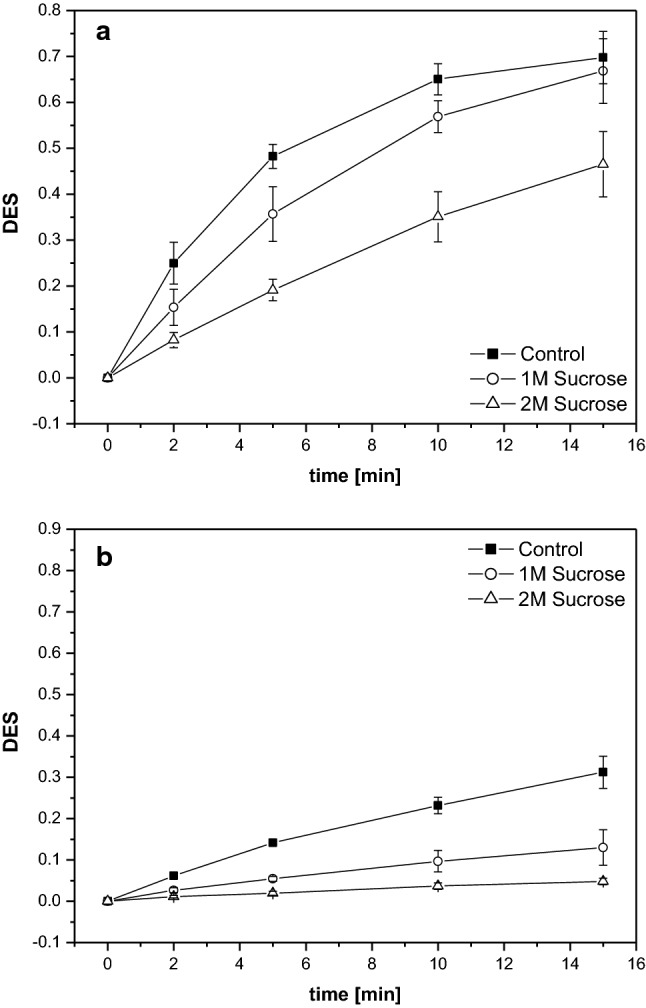
Time course of V de-epoxidation depicted as the de-epoxidation state of the V cycle pigment pool (DES). V de-epoxidation was performed at 30 °C (**a**) or 10 °C (**b**) in in vitro enzyme assays with isolated VDE in reaction buffers without or with sucrose. Purified V and MGDG were used with final concentrations of 0.4 and 11.6 µM, respectively, ascorbate was added at a final concentration of 30 mM. For further measurement conditions, including the pre-incubation conditions, see “Materials and methods”. Mean values of three independent measurements with the respective standard deviations are shown

The in vitro de-epoxidation assays which were performed with isolated VDE, purified V and MGDG show similar results to the de-epoxidation assays with the isolated thylakoid membranes. At both 30 °C (Fig. 5a) and 10 °C (Fig. 5b) a strong decrease of the conversion of V to A and Z can be seen in the presence of sucrose. Again the higher sucrose concentration leads to a more pronounced decrease of the de-epoxidation reaction.

**Fig. 6:**
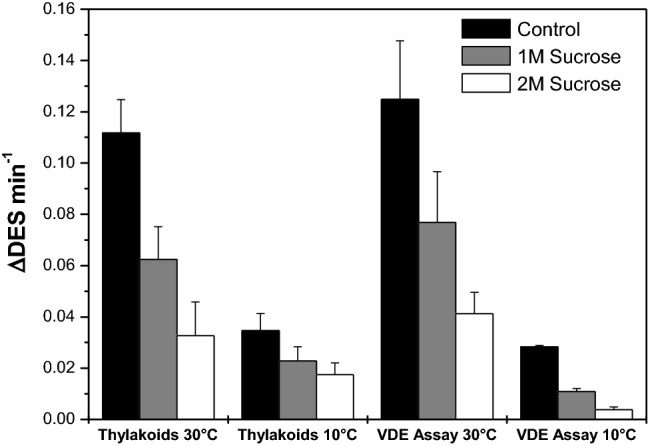
Dependence of the de-epoxidation rates on the concentration of the compatible solute sucrose in thylakoids or in vitro enzyme assays at 30 or 10 °C. The de-epoxidation rates are calculated as change of the DES per minute. The data used for Fig. 6 were derived from the DES shown in Figs. 4 and 5. For the calculation of the de-epoxidation rates at 30 °C the time point 2 min of the de-epoxidation reaction was used, the data for the measurements at 10 °C were calculated with the DES obtained after 5 min of the de-epoxidation assays. For further detail, see the legends of Figs. 4 and 5 and “Materials and methods”. Figure 6 shows the mean values of three independent measurements with the respective standard deviations

The de-epoxidation rates also show the strong inhibitory effect of sucrose on V conversion (Fig. 6). The de-epoxidation rates were calculated as change of the DES per minute from fixed time points of the de-epoxidation reaction and thus have to be considered semi-quantitative (for further explanation see the Materials and methods section and the legend of Fig. 6). In control thylakoids in the absence of sucrose at 30 °C a de-epoxidation rate of around 0.11 min^−1^ can be observed which drops to around 0.06 min^−1^ and 0.03 min^−1^ in the presence of 1 and 2 M sucrose, respectively. In the de-epoxidation assays performed with thylakoids without sucrose at the lower temperature of 10 °C a de-epoxidation rate of around 0.03 min^−1^ can be determined. The more than three-fold lower de-epoxidation rate compared to control thylakoids at 30 °C corresponds quite well with the expected decrease in enzyme activity caused by the temperature difference of 20 °C. At 10 °C the effect of the compatible solute again becomes visible as a significant decrease of the de-epoxidation rate. The results of the in vitro assays with isolated VDE, V and pure MGDG are similar to the experiments with the isolated thylakoids. Control enzyme assays in the absence of sucrose at 30 °C are characterized by a rate of around 0.13 min^−1^ which corresponds quite well to the de-epoxidation rate of the control thylakoid membranes at the higher temperature. This indicates that the VDE concentration (and thus activity) chosen for the in vitro enzyme assays was suitable for a direct comparison with the thylakoid experiments. Like in the de-epoxidation assays with thylakoids sucrose exerts a strong inhibitory effect on the conversion of V to A and Z. While for the enzyme assays at 30 °C a comparable reduction of the de-epoxidation rates to the thylakoid assays at 30 °C can be determined in the presence of sucrose, the inhibitory effect is more pronounced in the enzyme assays at 10 °C. With respect to the temperature effect we observe a slightly stronger reduction of the de-epoxidation rate upon lowering the temperature from 30 °C to 10 °C in the in vitro assays. However, the more than four-fold reduction of the de-epoxidation rate is also in line with the expected decrease in enzyme activity caused by a temperature difference of 20 °C.

**Fig. 7:**
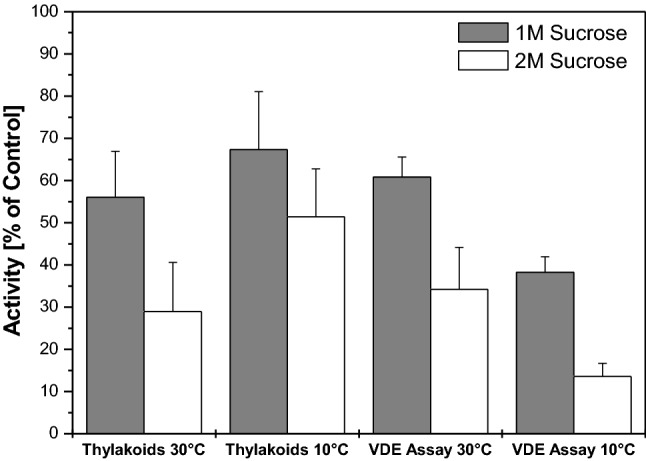
Dependence of V de-epoxidation on the concentration of the compatible solute sucrose in thylakoids or in vitro enzyme assays at 30 or 10 °C. Figure 7 depicts the de-epoxidation rates of de-epoxidation assays with thylakoid membranes or isolated VDE performed in the presence of sucrose as percentage of activity of the de-epoxidation rates of de-epoxidation assays with thylakoids or isolated VDE performed in reaction buffers without sucrose. The data used for Fig. 7 were derived from the de-epoxidation rates depicted in Fig. 6. For further detail see the legend of Fig. 6 and “Materials and methods”. Figure 7 shows the mean values of three independent measurements with the respective standard deviations

The depiction of the de-epoxidation rates of the sucrose-treated thylakoids or VDE in the enzyme assays as percentage of the de-epoxidation rates in the absence of sucrose (Fig. 7) allows a better comparison of the effect of sucrose on thylakoids and the isolated VDE. In the de-epoxidation assays with thylakoids at 30 °C a reduction of the de-epoxidation reaction of around 45 and 70% is observed for 1 and 2 M sucrose, respectively. The loss of activity in the enzyme assays at 30 °C is comparable to the loss of activity in the experiments with thylakoids at 30 °C. In the in vitro assays, 1 M of sucrose leads to a reduction of the de-epoxidation reaction of around 40% whereas 2 M sucrose decreases the de-epoxidation efficiency by about 65%. At 10 °C the results of the de-epoxidation assays with thylakoids and isolated VDE show differences. A stronger reduction of the conversion of V to A and Z in the presence of 1 and 2 M sucrose can be seen in the enzyme assays (around 60 and more than 80%, respectively) compared to the decrease of the de-epoxidation rate in thylakoids (around 35% in the de-epoxidation assays with 1 M sucrose and 50% in the assays with 2 M sucrose).

Summarizing the results of the V de-epoxidation assays it can be stated that the compatible solute sucrose inhibits the de-epoxidation reaction and that the magnitude of inhibition depends on the sucrose concentration. The inhibition of the conversion of V to A and Z by sucrose at 30 °C is comparable in de-epoxidation assays with either thylakoid membranes or isolated VDE and purified V and MGDG. At 10 °C the effect of sucrose seems to be more pronounced in the in vitro enzyme assays. The decreases of the de-epoxidation rates in both thylakoid and enzyme assays observed at the lower temperature are in line with the RGT-rule (or van’t Hoff-rule) which states that chemical reactions are increased by a factor of two or three when the temperature is increased by 10 K.

## Discussion

### Influence of sucrose on the organization of the membrane lipid phase

The results of the present study show that sucrose leads to changes of the organization of the lipid phase of thylakoid membranes and the order of membrane lipids. The effect of sucrose is visible at a temperature of 30 °C but not very pronounced. However, at 10 °C, the presence of sucrose maintains an organization of the membrane lipid phase which is comparable to that of membranes at 30 °C. In the present experiments the incubation of Laurdan with the thylakoid membranes was always carried out at a temperature of 30 °C to allow the complete incorporation of Laurdan into the membrane. It is possible that during the dilution of the thylakoids in the sucrose containing reaction buffers adjusted to a temperature of 10 °C sucrose is able to stabilize the organization of the lipid phase of the membrane usually present at higher temperatures. Stabilization of membranes by compatible solutes and sucrose has been described in various studies performed with artificial membrane systems composed of phospholipids (Uso and Rossignol 1984; Andersen et al. 2011; Laner et al. 2014). While at low concentrations stabilization is realized by the intercalation of sugar molecules between the lipid head groups and hydrogen bonding to the respective phosphate groups, high concentrations of compatible solutes, like those used for sucrose in the present study, lead to an exclusion of sugars from the membrane and the lipid-water interface. It is thought that the preferential exclusion increases the interfacial free energy and thus promotes the stability of lipid phases with low water accessibility. In the present experiments the sucrose-dependent changes of the Laurdan fluorescence and GP-values may have also been caused by lipid phase separation and extrusion of non-bilayer lipid phases from the membrane bilayer. This would be in line with the early observations by Williams et al. (1992) who used freeze-fracture electron microscopy to show that high concentrations of compatible solutes lead to a phase separation of the thylakoid bilayer and non-bilayer lipids and thus the formation of rather extensive non-bilayer lipid phases. Recent results based on ^31^P-NMR spectroscopy and time-resolved fluorescence emission spectra of the fluorescent dye merocyanine-540 indicate that one type of bilayer and three types of non-bilayer phases co-exist in the thylakoid membrane (Garab et al. 2017). Using the same methods Kotakis et al. (2018) analysed in closer detail the effects of 2 M sucrose on the lipid structure of isolated thylakoid membranes and observed a separation of bilayer and non-bilayer lipid phases by the presence of the compatible solute. Since in the present experiments the effects of sucrose are very pronounced at a temperature of 10 °C this would indicate that an extensive phase separation accompanied by the formation of large non-bilayer phases takes place at the low temperature. At 30 °C, only minor effects of sucrose on the membrane organization are visible which would argue for only a slight increase of the non-bilayer phases at the high temperature. These differences in the effect of sucrose could be explained if the thylakoid membranes at 30 °C per se are characterized by the presence of large areas of non-bilayer lipid phases whereas in membranes at 10 °C the percentage of H_II_-phases is significantly reduced. In addition, it has to be taken into account that Garab et al. (2017) demonstrated that low pH values lead to a marked increase of the non-bilayer phases within the thylakoid membrane because, in the experiments of the present study, pH values of 5 were used to induce the enzymatic de-epoxidation of V to A and Z.

We suggest that for the interpretation of the present results it has to be taken into account that the native thylakoid membrane contains high concentrations of non-bilayer lipids and membrane proteins and that the membrane organization may differ at low and high temperatures. It is likely that the change of membrane organization observed in the presence of sucrose reflects the complex situation in the native membrane which includes the simultaneous presence of several lipid bilayer and non-bilayer phases. High concentrations of sucrose may also influence the thylakoid membrane proteins and thus change the lipid protein interactions and the ratio of bilayer to non-bilayer lipid phases. This interpretation is supported by our finding that in the in vitro enzyme assays, where MGDG is used as a single lipid and where no membrane proteins are present, both sucrose and temperature have no or only a very low impact on the GP-values of the Laurdan fluorescence. This indicates that the inverted hexagonal phase, which is most likely formed by MGDG in these assays, is stable in the temperature range used for the present experiments, i.e. 10 to 30 °C. In addition, high concentrations of sucrose, which change the organization of the complex thylakoid membrane with its different bilayer and non-bilayer phases and incorporated membrane proteins do not affect the structure and lipid order of the single non-bilayer MGDG phase in the enzyme assays.

### Influence of sucrose on V de-epoxidation

The present results show that sucrose inhibits the de-epoxidation of V to A and Z. This is unexpected because compatible solutes like sucrose should lead to an extensive phase separation of the thylakoid membrane lipids and thus to the formation of large non-bilayer lipid phases (Williams et al. 1992). Since various results have pointed to an importance of non-bilayer phases for the process of V de-epoxidation (Latowski et al. 2004; Goss et al. 2005, 2007), a more efficient conversion of V to A and Z was expected in the presence of sucrose. The decrease in V de-epoxidation in the presence of sucrose is puzzling since the slight changes at 30 °C and the marked differences of thylakoid membrane organization in the presence of sucrose at 10 °C indicate an increased lipid phase separation and formation of non-bilayer lipid phases. However, the sucrose-dependent membrane re-organization does not exert a positive effect on the conversion of V to Z. This is especially obvious at the temperature of 10 °C where the amount of membrane re-organization increases with increasing sucrose concentration while the efficiency of V de-epoxidation decreases. Taking into account the concept of membrane stabilization by sucrose, the organization of the membrane lipid phase at 10 °C in the presence of sucrose should be comparable to that at 30 °C and thus allow a better conversion of V to A and Z. However, this is not the case and the V de-epoxidation is even slower than the strongly reduced de-epoxidation of control thylakoids without sucrose at 10 °C.

The decreased GP-values in the presence of sucrose may also indicate an increased membrane fluidity in the presence of the compatible solute which is especially obvious in the thylakoid membranes exposed to the low temperature of 10 °C. Note, however, that in complex membranes such as the thylakoid membrane a direct relation between GP-values and membrane fluidity is questionable (see also last paragraph of the Introduction section). According to Arvidsson et al. (1997) and Macko et al. (2002), who see the diffusion of V from the LHCII to the non-bilayer lipid phase as the rate limiting step of the de-epoxidation reaction, this should have a positive effect on V de-epoxidation. However, in the present experiments the addition of sucrose leads to a strong reduction of the V conversion despite the positive effects on membrane organization and possibly membrane fluidity. It seems that under the conditions of the present study the overall organization of the isolated thylakoid membranes does not have a pronounced impact on the V de-epoxidation reaction.

This is corroborated by the results of the in vitro enzyme assays where the organization and lipid order of the inverted hexagonal MGDG phase is unaffected by both the temperature and the presence of sucrose. However, comparable decreases of the V de-epoxidation rate to the experiments with isolated thylakoid membranes can be observed upon the addition of 1 and 2 M sucrose. The in vitro experiments indicate that sucrose exerts a direct effect on the activity of the VDE. This is in line with several studies (Kendrick et al. 1997; Timasheff 2002; Kim et al. 2003; Street et al. 2006) which have shown that compatible solutes increase the thermodynamic conformational stability of proteins, thus stabilizing the native structure of the protein. This might be beneficial for membrane proteins such as PSII, whose temperature-dependent inactivation is decreased in the presence of sucrose (Williams et al. 1992; Kotakis et al. 2018). However, the increased protein stability might have a negative effect on enzymatic reactions which require dynamic interaction between the catalytic site and the substrate, such as the VDE. With regard to the last aspect, it was demonstrated that an increased viscosity of the medium, as it is induced by compatible solutes, results in a strong reduction of the catalytic rate of enzymes which undergo large conformational changes during the catalytic reaction (Demchenko et al. 1989; Uribe and Sampedro 2003). It is also possible that the decreased water availability in the presence of high sucrose concentrations removes at least part of the hydrate shell of the VDE. For the VDE this implies, that those parts of the enzyme which, after binding to the thylakoid membrane or the inverted hexagonal MGDG phase in the enzyme assays, protrude from the non-bilayer lipid phase are exposed to the increased viscosity of the medium or the decreased water availability. Stabilization of the respective protein parts or removal of part of the hydrate shell might then have an impact on the catalytic site and decrease the catalytic activity. The localization of the exposed VDE parts, which interact with sucrose, is presently unclear. According to our knowledge, no sucrose transporters have been described for the thylakoid membrane which could enable sucrose transport over the intact membrane and thus realize higher sucrose concentrations in the thylakoid lumen. It is also not clear if substantial amounts of sucrose are able to diffuse from the stromal side of the membrane into the lumen. This means that the question if sucrose interacts with parts of the VDE that are exposed at the stromal or the luminal side of the non-bilayer lipid phase of the thylakoid membrane has to remain open. While it is clear that VDE binds at the luminal side of the non-bilayer lipid phase after activation (Hager and Holocher 1994), the size of the active, dimeric VDE would certainly allow a complete penetration of the inverted hexagonal phase and thus the protrusion of certain parts of the enzyme at the stromal side (for the molecular weight of monomeric VDE see Arvidsson et al. 1996). In addition, recent results have indicated that both the N-terminal as well as the C-terminal region of the VDE contain *α*-helical structures (Hallin et al. 2016). While these domains are important for enzyme activity and pH-dependent solubility they may also play a role in the interaction with the lipid phase. Finally, the stronger inhibition of V de-epoxidation in the enzyme assays at 10 °C compared to the assays with thylakoids at the same temperature indicates that in the MGDG phase of the enzyme assays larger areas of the VDE are exposed to the sucrose containing medium than in the putative non-bilayer lipid phase of the thylakoid membranes.

#### *Author contribution statement*

RG conceived and designed research, analysed data, wrote manuscript. CS, MM designed research, conducted experiments, analysed data, corrected manuscript. CW analysed data, corrected manuscript. All authors read and approved the manuscript.

## Data Availability

All data supporting the findings of this study are available within the article. Additional data sets generated during and/or analysed during the current study are available from the corresponding author upon reasonable request.

## Abbreviations

A: Antheraxanthin
DES: De-epoxidation state of the violaxanthin cycle pigment pool
GP: Generalized polarization
H_II_: Phase-inverted hexagonal phase
LHC: Light-harvesting complex
MGDG: Monogalactosyldiacylglycerol
V: Violaxanthin
VDE: Violaxanthin de-epoxidase
Z: Zeaxanthin
ZEP: Zeaxanthin epoxidase

## References

[CR1] Andersen HD, Wang C, Arleth L, Peters GH, Westh P (2011). Reconciliation of opposing views on membrane-sugar interactions. Proc Natl Acad Sci USA.

[CR2] Arnoux P, Morosinotto T, Saga G, Bassi R, Pignol D (2009). A structural basis for the pH-dependent xanthophyll cycle in *Arabidopsis thaliana*. Plant Cell.

[CR3] Arvidsson P-O, Bratt CE, Carlsson M, Akerlund H-E (1996). Purification and identification of the violaxanthin deepoxidase as a 43 kDa protein. Photosynth Res.

[CR4] Arvidsson P-O, Carlsson M, Stefansson H, Albertsson PA, Akerlund H-E (1997). Violaxanthin accessibility and temperature dependency for de-epoxidation in spinach thylakoid membranes. Photosynth Res.

[CR5] Böhme K, Wilhelm C, Goss R (2002). Light regulation of carotenoid biosynthesis in the prasinophycean alga *Mantoniella squamata*. Photochem Photobiol Sci.

[CR205] Demchenko AP, Rusyn OI, Saburova EA (1989). Kinetics of lactate dehydrogenase reaction in high viscosity media. Biochim Biophys Acta.

[CR6] Frommolt R, Goss R, Wilhelm C (2001). The de-epoxidase and epoxidase reactions of *Mantoniella squamata* (Prasinophyceae) exhibit different substrate-specific reaction kinetics compared to spinach. Planta.

[CR7] Garab G, Ughy B, Goss R, Nakamura Y, Li-Beisson Y (2016). Role of MGDG and non-bilayer lipid phases in the structure and dynamics of chloroplast thylakoid membranes. Lipids in plant and algae development.

[CR8] Garab G, Ughy B, de Waard P, Akhtar P, Javornik U, Kotakis C, Sket P, Karlicky V, Materova Z, Spunda V, Plavec J, van Amerongen H, Vigh L, Van As H, Lambrev PH (2017). Lipid polymorphism in chloroplast thylakoid membranes—as revealed by ^31^P-NMR and time resolved merocyanine fluorescence spectroscopy. Sci Rep.

[CR9] Goss R (2003). Substrate specificity of the violaxanthin de-epoxidase of the primitive green alga *Mantoniella squamata* (Prasinophyceae). Planta.

[CR10] Goss R, Latowski D (2020). Lipid dependence of xanthophyll cycling. Front Plant Sci.

[CR11] Goss R, Lepetit B (2015). Biodiversity of NPQ. J Plant Physiol.

[CR12] Goss R, Lohr M, Latowski D, Grzyb J, Vieler A, Wilhelm C, Strzalka K (2005). Role of hexagonal structure-forming lipids in diadinoxanthin and violaxanthin solubilization and de-epoxidation. Biochemistry.

[CR13] Goss R, Latowski D, Grzyb J, Vieler A, Lohr M, Wilhelm C, Strzalka K (2007). Lipid dependence of diadinoxanthin solubilization and de-epoxidation in artificial membrane systems resembling the lipid composition of the natural thylakoid membrane. Biochim Biophys Acta.

[CR14] Goss R, Greifenhagen A, Bergner J, Volke D, Hoffmann R, Wilhelm C, Schaller-Laudel S (2017). Direct isolation of a functional violaxanthin cycle domain from thylakoid membranes of higher plants. Planta.

[CR15] Gounaris K, Brain APR, Quinn PJ, Williams WP (1984). Structural reorganization of chloroplast thylakoid membranes in response to heat stress. Biochim Biophys Acta.

[CR16] Hager A, Holocher K (1994). Localization of the xanthophyll-cycle enzyme violaxanthin de-epoxidase within the thylakoid lumen and abolition of its mobility by a (light-dependent) pH decrease. Planta.

[CR17] Hallin EI, Hasan M, Guo K, Akerlund H-E (2016). Molecular studies on structural changes and oligomerisation of violaxanthin de-epoxidase associated with the pH-dependent activation. Photosynth Res.

[CR18] Harris FM, Best KB, Bell JD (2002). Use of Laurdan fluorescence intensity and polarization to distinguish between changes in membrane fluidity and phospholipid order. Biochim Biophys Acta.

[CR19] Hellgren LI (1996). A novel method in studies of dynamical properties in plant membranes: laurdan fluorescence spectroscopy. Plant Physiol Biochem.

[CR20] Hieber AD, Bugos RC, Yamamoto HY (2000). Plant lipocalins: violaxanthin de-epoxidase and zeaxanthin epoxidase. Biochim Biophys Acta.

[CR21] Jahns P, Latowski D, Strzalka K (2009). Mechanism and regulation of the violaxanthin cycle: the role of antenna proteins and membrane lipids. Biochim Biophys Acta.

[CR22] Jensen RG, Bassham JA (1966). Photosynthesis by isolated chloroplasts. Proc Natl Acad Sci USA.

[CR200] Kendrick BS, Chang BS, Arakawa T, Peterson B, Randolph TW, Manning MC, Carpenter JF (1997). Preferential exclusion of sucrose from recombinant interleukin-1 receptor antagonist: role in restricted conformational mobility and compaction of native state. Proc Natl Acad Sci USA.

[CR203] Kim Y-S, Jones LS, Dong A, Kendrick BS, Chang BS, Manning MC, Randolph TW, Carpenter JF (2003). Effects of sucrose on conformational equilibria and fluctuations within the native-state ensemble of proteins. Protein Sci.

[CR23] Kotakis C, Akhtar P, Zsiros O, Garab G, Lambrev PH (2018). Increased thermal stability of photosystem II and the macro-organization of thylakoid membranes, induced by co-solutes, associated with changes in the lipid-phase behaviour of thylakoid membranes. Photosynthetica.

[CR24] Laner M, Horta BAC, Hünenberger PH (2014). Effect of the cosolutes trehalose and methanol on the equilibrium and phase-transition properties of glycerol-monopalmitate lipid bilayers investigated using molecular dynamics simulations. Eur Biophys J.

[CR25] Latowski D, Akerlund H-E, Strzalka K (2004). Violaxanthin de-epoxidase, the xanthophyll cycle enzyme, requires lipid inverted hexagonal structures for its activity. Biochemistry.

[CR26] Liu Z, Yan H, Wang K, Kuang T, Zhang J, Gui L, Chang W (2004). Crystal structure of spinach major light-harvesting complex at 2.72 Å resolution. Nature.

[CR27] Macko S, Wehner A, Jahns P (2002). Comparison of violaxanthin de-epoxidation from the stroma and lumen sides of isolated thylakoid membranes from *Arabidopsis*: implications for the mechanism of de-epoxidation. Planta.

[CR28] Parasassi T, De Stasio G, d’Ubaldo A, Gratton E (1990). Phase fluctuation in phospholipid membranes revealed by Laurdan fluorescence. Biophys J.

[CR29] Parassasi T, De Stasio G, Ravagnan G, Rusch RM, Gratton E (1991). Quantitation of lipid phases in phospholipid vesicles by the generalized polarization of Laurdan fluorescence. Biophys J.

[CR30] Parasassi T, Krasnowska EK, Bagatolli L, Gratton E (1998). Laurdan and Prodan as polarity-sensitive fluorescent membrane probes. J Fluoresc.

[CR31] Pfündel EE, Renganathan M, Gilmore AM, Yamamoto HY, Dilley RA (1994). Intrathylakoid pH in isolated pea chloroplasts as probed by violaxanthin deepoxidation. Plant Physiol.

[CR32] Ruban AV (2016). Nonphotochemical fluorescence quenching: mechanism and effectiveness in protecting plants from photodamage. Plant Physiol.

[CR33] Schaller S, Latowski D, Jemiola-Rzeminska M, Wilhelm C, Strzalka K, Goss R (2010). The main thylakoid membrane lipid monogalactosyldiacylglycerol (MGDG) promotes the de-epoxidation of violaxanthin associated with the light-harvesting complex of photosystem II (LHCII). Biochim Biophys Acta.

[CR34] Schaller S, Wilhelm C, Strzalka K, Goss R (2012). Investigating the interaction between the violaxanthin cycle enzyme zeaxanthin epoxidase and the thylakoid membrane. J Photochem Photobiol b: Biology.

[CR204] Street TO, Bolen DW, Rose GD (2006). A molecular mechanism for osmolyte-induced protein stability. Proc Natl Acad Sci USA.

[CR35] Szilagyi A, Selstam E, Akerlund H-E (2008). Laurdan fluorescence spectroscopy in the thylakoid bilayer: the effect of violaxanthin to zeaxanthin conversion on the galactolipid dominated lipid environment. Biochim Biophys Acta.

[CR201] Timasheff SN (2002). Protein-solvent preferential interactions, protein hydration, and the modulation of biochemical reactions by solvent components. Proc Natl Acad Sci USA.

[CR36] Thomas PG, Brain APR, Quinn PJ, Williams WP (1985). Low pH and phospholipase A_2_ treatment induce the phase separation of non-bilayer lipids within pea chloroplast membranes. FEBS Letts.

[CR206] Uribe S, Sampedro JG (2003). Measuring solution viscosity and its effect on enzyme activity. Biol Proceed Online.

[CR37] Uso T, Rossignol M (1984). Sucrose effects on the fluidity of natural and synthetic phospholipid bilayers. FEBS Lett.

[CR38] Williams WP, Brain APR, Dominy PJ (1992). Induction of non-bilayer lipid phase separations in chloroplast thylakoid membranes by compatible co-solutes and its relation to the thermal stability of photosystem II. Biochim Biophys Acta.

[CR39] Yamamoto HY, Higashi RM (1978). Violaxanthin de-epoxidase. Lipid composition and substrate specificity. Arch Biochem Biophys.

